# Asymptomatic hyponatremia: is it time to abandon this entity?

**Published:** 2015-03-10

**Authors:** Manjunath Kulkarni, Archana Bhat

**Affiliations:** Department of Nephrology, Father Muller Medical College, Mangalore, Karnataka, India; Department of Pathology, Father Muller Medical College, Mangalore, Karnataka, India

**Keywords:** Hyponatremia, Osteoporosis, Mortality

**Implication for health policy/practice/research/medical education:**

Hyponatremia is a commonly encountered electrolyte abnormality in clinical practice. It has been common practice not to evaluate or treat so called ‘asymptomatic hyponatremia.’ However it is being increasingly recognized that even mild degrees of hyponatremia are not benign. Mild hyponatremia is associated with subtle changes in gait and associated with increased risk of fractures. The purpose of this paper is to draw attention of clinicians to this condition. Even mild asymptomatic hyponatremia requires evaluation and treatment .

## Introduction

Hyponatremia is the most common electrolyte abnormality seen in clinical practice. It occurs in wide variety of clinical conditions. Studies have shown that presence of hyponatremia is a risk factor for morbidity and mortality. Despite hyponatremia being a commonly encountered condition, there is no consensus in its management. Recently published guidelines have made an attempt to bring uniformity in management of hyponatremia. There is a consensus that all symptomatic hyponatremia must be treated. The purpose of this paper is to question the existence of ‘asymptomatic hyponatremia.’ Analogy is similar to hypokalemia wherein even milder degree of hypokalemia is treated because of risk of cardiac arrhythmia. But most of the clinicians are ready to accept mild degree of hyponatremia as harmless.

Hyponatremia is defined as serum sodium of less than 135 mEq/l. It is the most common abnormality of fluid and electrolytes encountered in clinical practice. It occurs in up to 30% of hospitalized patients and can lead to a wide spectrum of clinical symptoms, from subtle to severe or even life threatening (1). It can be classified on basis of duration, severity and symptoms (Box 1). Traditional teaching is to classify hyponatremia as symptomatic and asymptomatic based on symptoms. Based on recent studies the very existence of ‘asymptomatic hyponatremia’ needs to question.

## Does asymptomatic hyponatremia really exist?

Symptoms of hyponatremia can vary from mild, non-specific to severe and life-threatening. Severe symptoms are because of cerebral edema and increased intracranial pressure. Severe signs of acute hyponatremia are well established and can be easily recognized by the clinicians. It is of utmost importance to understand the fact that even patients with chronic hyponatremia and no apparent symptoms can have subtle clinical abnormalities (Box 2). The subtle effects may be go undetected on a casual clinical examination.

**Box 1. Classification of hyponatremia**

1. Based on duration: Acute or chronic based on whether hyponatremia has developed over less than or more than 48 hours.
2. Based on biochemical values: Mild (130-135 mEq/l), moderate (125-129 mEq/l) and severe (<125 mEq/l).
3. Based on symptoms: It is usually divided into symptomatic and asymptomatic. On the basis of recent understanding and studies it is better to classify hyponatremia as moderately symptomatic or severely symptomatic. It would be preferable to avoid the term asymptomatic hyponatremia.

**Box 2. Symptoms of chronic hyponatremia**

- Fatigue
- Nausea
- Dizziness
- Gait disturbances
- Forgetfulness
- Confusion
- Lethargy
- Muscle cramps

## Subtle effects of hyponatremia

Renneboog et al studied 16 patients with mild chronic hyponatremia (mean serum sodium 128 mg/dl) who had a normal neurological examination and were considered ‘asymptomatic’ clinically. All had a mini-mental state examination (MMSE) score of 29 or 30. These patients were subjected to attention, posture assessment and gait tests. In attention tests, mean response time was significantly higher in hyponatremic patients as compared to controls. The latency in chronic hyponatremia was higher than controls after alcohol consumption. The effects of hyponatremia on attention tests were more than that of alcohol. The same study also found that there was significant balance impairment in patients with chronic hyponatremia and the effect was worse than alcohol consumption (2).

In a prospective study of 56 chronic dialysis patients, it was found that mild hyponatremia was associated with functional and cognitive decline (3).

## Hyponatremia and risk of fall and fractures

Recent studies have highlighted the importance of hyponatremia and risk of fall and fractures especially in the elderly. In general, prevalence of hyponatremia in patients with femoral fracture was 19.5% (4). Prevalence of hyponatremia in elderly patients with fragility fractures was found to be even higher. About 26% in elderly patients with fragility fractures were found to have hyponatremia (5). Accordingly, in a retrospective analysis of 543 patients with femoral fracture compared to 700 outpatient controls, it was found that hyponatremia was significantly associated with fracture. The odds ratio of hyponatremia for femoral neck fracture was 2.08 in the study population (1). In a study 2370 geriatric trauma patients, hyponatremia was significantly associated with admission for a fall than non-hyponatremics. (6). Moreover, Renneboog et al found that frequency of falls was 21% in hyponatremics as compared to 5.3% in normonatremics. They also found that after controlling for age, sex and other known risk factors for falls, the adjusted odds ratio for falls with hyponatremia was 67 compared with controls (1).

Studies have concluded that identification and management of hyponatremia should be an integral part of fall prevention program. To prevent further episodes of fall, correction of hyponatremia before discharge from hospital is mandatory (4,6).

## Hyponatremia and osteoporosis

Analysis of NHANES 3 also found that the adjusted odds of osteoporosis were significantly higher among participants with hyponatremia than among those with normonatremia (7). Hyponatremia increases risk of osteoporosis at both hip and lumbar spine. The study even concluded that hyponatremia can be used as a screening tool and marker of osteoporosis (8).

## Hyponatremia and mortality

A prospective study of 98411 hospitalized patients found that hyponatremia even mild is associated with increased mortality (9). Another study found that after hospitalization one year mortality was 27.5% in hyponatremic patients as compared to 17.7% in normonatremics. Hyponatremia was associated with longer duration of stay in hospital (10). Patients with acute myocardial infection and hyponatremia on admission showed a significantly higher risk of mortality than patients without this abnormality (11).

## Conclusion

Hyponatremia even mild is not a benign entity. It has effect on cognition, balance and attention. It increases risk of falls and fractures. Apart from association with mortality, it increases risk of osteoporosis. Clinicians should make every attempt to identify and treat even milder forms of hyponatremia.

## Authors’ contribution

MK; concept, design, data collection, drafting, and editing. AB; design, data collection, drafting, and data analysis.

## Conﬂicts of interest

The authors declared no competing interests.

## Ethical considerations

Ethical issues (including plagiarism, data fabrication, double publication) have been completely observed by the authors.

## Funding/Support

None.
